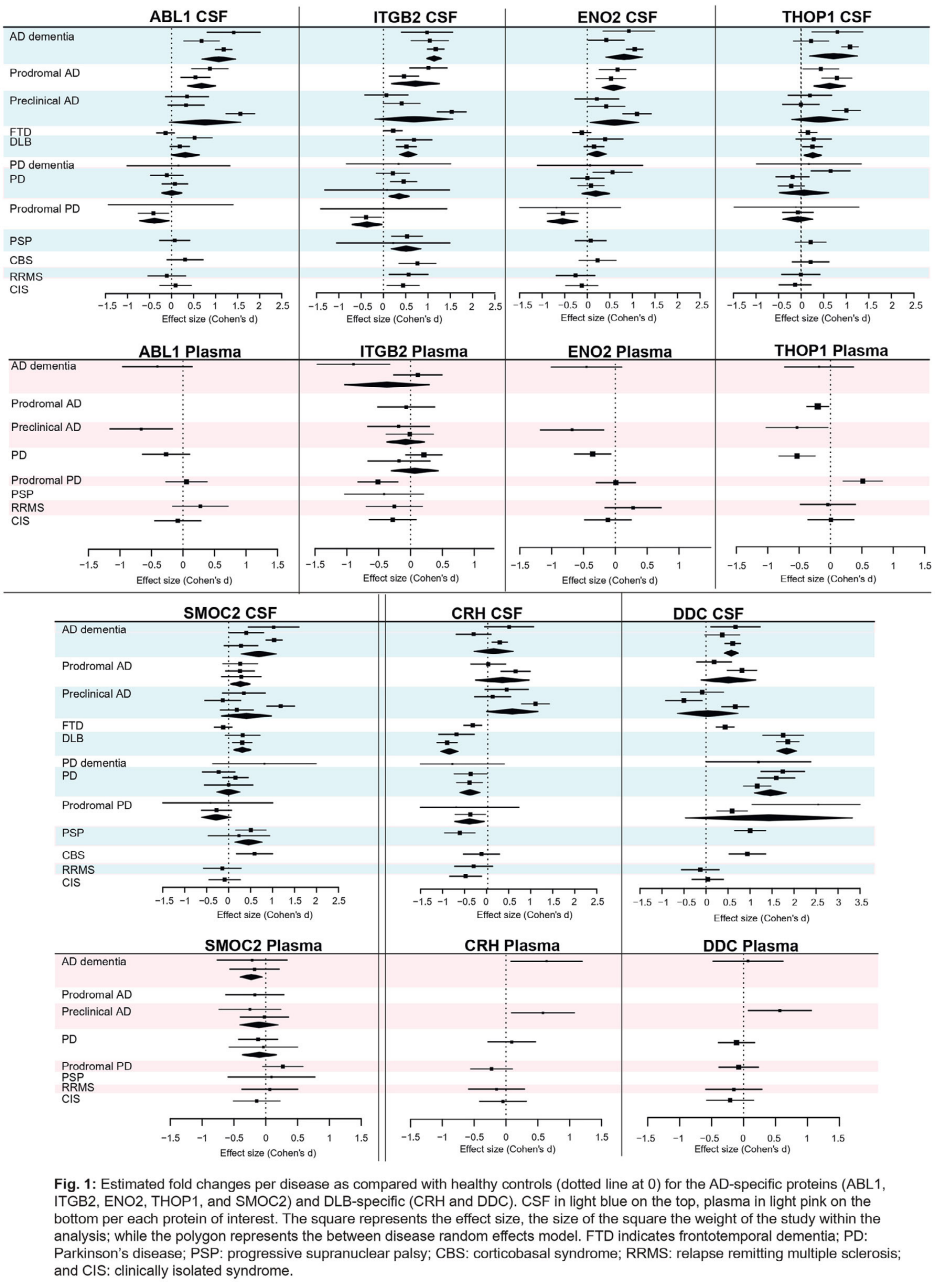# Biofluid biomarkers across neurological disorders: a systematic meta‐analysis of shared and unique molecular signatures

**DOI:** 10.1002/alz70856_105820

**Published:** 2026-01-07

**Authors:** Elena Raluca Blujdea, Yanaika S. Hok‐A‐Hin, Kira Trares, Marta del Campo, Charlotte E. Teunissen, Lisa Vermunt

**Affiliations:** ^1^ Neurochemistry Laboratory, Department of Laboratory Medicine, Vrije Universiteit Amsterdam, Amsterdam UMC, Amsterdam, Netherlands; ^2^ Neurochemistry Laboratory, Department of Laboratory Medicine, Amsterdam Neuroscience, Vrije Universiteit Amsterdam, Amsterdam UMC, Amsterdam, Netherlands; ^3^ German Cancer Research Center, Heidelberg, Baden‐Wuerttemberg, Germany; ^4^ Hospital del Mar Research Institute (IMIM), Barcelona, Spain; ^5^ Barcelonaβeta Brain Research Center (BBRC), Pasqual Maragall Foundation, Barcelona, Spain; ^6^ Neurochemistry Laboratory, Department of Laboratory Medicine, Amsterdam UMC, Vrije Universiteit Amsterdam, Amsterdam Neuroscience, Amsterdam, Netherlands; ^7^ Neurochemistry Laboratory, Department of Laboratory Medicine, Vrije Universiteit Amsterdam, Amsterdam UMC location VUmc, Amsterdam, Netherlands

## Abstract

**Background:**

A comprehensive analysis of proximity‐extension assay (PEA) proteomic studies have led to the development and validation of novel cerebrospinal fluid (CSF) biomarker panels for Alzheimer's disease (AD; Del Campo et al., 2022 *Nat Aging*) and dementia with Lewy bodies (DLB; Del Campo et al., 2023 *Nat Comm*.). However, to what extent the markers are dementia‐ and matrix‐ (CSF or plasma) specific is largely unknown. To establish the clinical context for these novel markers, data from PEA proteomic studies in CSF and plasma will be collected and meta‐analyzed across neurological disorders.

**Methods:**

A systematic review following PRISMA‐guidelines is being conducted to collect relevant CSF and plasma PEA studies. For six CSF and plasma studies with readily available data in dementia and movement disorders, Cohen's *d* effect sizes were calculated for seven proteins identified as AD‐specific (ABL1, ITGB2, ENO2, SMOC2, and THOP1) or DLB‐specific (DDC and CRH) – measuring the mean difference between disease and control groups. A random‐effects meta‐analysis model was applied within the same disorders and plotted, Figure 1.

**Results:**

Analysis showed that for all seven proteins, when comparing effect sizes between CSF and plasma, the direction of change is opposite. This suggests that these markers may only be useful in CSF, and in plasma may even associate with other disorders; as THOP1 seems to be most increased in prodromal PD, Figure 1. Within‐disease analysis revealed variability mainly in preclinical AD, for ABL1, ITGB2, and THOP1, and prodromal PD for DDC, supporting the biological heterogeneity of these groups. Additionally, DDC and CRH in CSF are distinctly dysregulated for Parkinson's‐plus syndromes (PD, DLB, PSP, CBS) highlighting the overlapping biological mechanisms.

**Conclusions:**

Our meta‐analysis across multiple cohorts is expanding to include around 150 studies in dementias, movement disorders and in psychiatric conditions. The framework of analysis established in this study provides a foundation for future expansion of the database in various neurological disorders, such as psychiatric disorders, and to other proteomic platforms. These findings may contribute to the development of more accurate diagnostic tools and offer insights into the complex interplay of protein markers in neurodegenerative diseases.